# Schizophrenia copy number variants and associative learning

**DOI:** 10.1038/mp.2016.227

**Published:** 2016-12-13

**Authors:** N E Clifton, A J Pocklington, B Scholz, E Rees, J T R Walters, G Kirov, M C O'Donovan, M J Owen, L S Wilkinson, K L Thomas, J Hall

**Affiliations:** 1 Neuroscience and Mental Health Research Institute, Cardiff University, Cardiff, UK; 2 MRC Centre for Neuropsychiatric Genetics and Genomics, Institute of Psychological Medicine and Clinical Neurosciences, Cardiff University, Cardiff, UK; 3 School of Psychology, Cardiff University, Cardiff, UK; 4 School of Biosciences, Cardiff University, Cardiff, UK

## Abstract

Large-scale genomic studies have made major progress in identifying genetic risk variants for schizophrenia. A key finding from these studies is that there is an increased burden of genomic copy number variants (CNVs) in schizophrenia cases compared with controls. The mechanism through which these CNVs confer risk for the symptoms of schizophrenia, however, remains unclear. One possibility is that schizophrenia risk CNVs impact basic associative learning processes, abnormalities of which have long been associated with the disorder. To investigate whether genes in schizophrenia CNVs impact on specific phases of associative learning we combined human genetics with experimental gene expression studies in animals. In a sample of 11 917 schizophrenia cases and 16 416 controls, we investigated whether CNVs from patients with schizophrenia are enriched for genes expressed during the consolidation, retrieval or extinction of associative memories. We show that CNVs from cases are enriched for genes expressed during fear extinction in the hippocampus, but not genes expressed following consolidation or retrieval. These results suggest that CNVs act to impair inhibitory learning in schizophrenia, potentially contributing to the development of core symptoms of the disorder.

## Introduction

Schizophrenia is a highly heritable psychiatric disorder characterised by psychotic, cognitive and negative symptoms.^1^ Since the disorder was first named by Bleuler in the early 20th century, abnormal association formation has been considered to represent a core underlying feature of the condition.^2^ However, the overall relationship between genetic risk for schizophrenia and alterations in association formation in specific tasks such as fear learning has not been well established.

In recent years major progress has been made in identifying genetic variants contributing to risk for schizophrenia, which include multiple common alleles of small effect and a significant component of rarer but more penetrant variants.^3, 4^ In particular, genomic studies have revealed a significant increased burden of rare chromosomal copy number variants (CNVs), which involve the deletion or duplication of thousands of bases of DNA, in schizophrenia patients compared with controls.^4, 5, 6, 7^ Pathway analysis of genes affected by these CNVs has revealed an enrichment for genes implicated in processes involved in synaptic plasticity, including genes encoding components of the NMDA receptor complex and post-synaptic density.^8, 9^ A key function of these synaptic proteins is to regulate the molecular processes underlying associative learning and memory.^10, 11, 12^ These findings are thus consistent with documented deficits in associative learning in patients with schizophrenia, which have been considered to contribute to the development and persistence of cognitive and psychotic symptoms.^13, 14, 15, 16, 17, 18^ However, it is not currently clear which learning processes are affected by schizophrenia CNVs.

The molecular basis of associative learning has been investigated in detail in animal models, in particular, in relation to contextual fear memory processing in the hippocampus.^19^ These studies have demonstrated that distinct molecular changes accompany the consolidation, retrieval and extinction of contextual fear associations^20, 21^ and that each of these stages of fear memory processing is accompanied by *de novo* gene expression.^22, 23^ Notably, memory extinction is recognised to be an active form of inhibitory learning that serves to form a new secondary, stimulus-no event association that competes with the original memory for control over behaviour.^24^ Abnormalities in extinction learning and related forms of inhibitory learning have been reported in patients with schizophrenia and may contribute to the development and persistence of psychotic and cognitive symptoms in the condition.^15, 25, 26^ It is not known, however, which components of associative learning are affected by genes in schizophrenia-associated CNVs.

Here, we have applied an integrated approach combining human genetics, bioinformatics and experimental studies in animals to investigate the hypothesis that genes in schizophrenia-associated CNVs impact on molecular pathways engaged during specific phases of associative learning. Specifically, we investigated whether CNVs in schizophrenia cases from three large cohorts are selectively enriched for genes expressed during the consolidation, retrieval or extinction of fear associations in the hippocampus. Identifying which specific cognitive processes are impacted by schizophrenia risk genes is an important step towards determining how genetic risk can lead to the development of the complex symptoms seen in schizophrenia and related disorders.

## Materials and methods

### CNV samples and quality control

CNV data were compiled from three European case-control data sets: the International Schizophrenia Consortium (ISC; 3395 cases, 3185 controls), the Molecular Genetics of Schizophrenia (MGS; 2215 cases, 2556 controls) study and a UK study of schizophrenic patients taking clozapine combined with the Cardiff Cognition in Schizophrenia sample (CLOZUK; 6307 cases, 10 675 controls),^5, 27, 28^ together, giving a total of 11 917 case and 16 416 control subjects. These CNV data sets have been previously validated and full genotyping, CNV calling and quality control information can be found in the original publications.^4, 5, 8, 27, 28^ Approval by the local ethics committee was granted for the use of these samples in genetic association studies. All CNVs from the ISC, MGS and CLOZUK data sets were annotated with their overlapping genes from corresponding genome Builds 35, 36 and 37, respectively. Analyses were performed on CNVs at least 100 kb in size and covered by at least 15 probes, to optimise CNV calling reliability. For comparison analyses, CNVs were compared between melanoma patients and additional non-psychiatric phenotypes. These CNVs were obtained from a separate study^29^ and underwent the same filtering process (2416 melanoma cases and 6335 additional controls).

### Consolidation, retrieval and extinction of contextual fear associations

Associative learning was assessed in a previously published set of experiments using hippocampus-dependent contextual fear conditioning in rats.^19^ Conditioning was characterised by robust conditioned freezing (immobility) responses upon re-exposure to the training environment indicating robust contextual fear conditioning and recall of contextual fear memory.^20, 21^ For a full description of the conditioning procedures and tissue collection see Barnes *et al.* (2012)^20^ and Scholz *et al.* (2016).^21^ In all experiments adult male Lister Hooded rats were placed into a context for 3 min during which they received a single delivery of a single-scrambled foot shock (0.5 mA, 2 s). We have previously shown that this context-shock pairing is sufficient to produce reliable contextual fear conditioning.^30, 31^ Rats were then killed 2 h after either consolidation, retrieval or extinction of the fear association. For the consolidation group, animals were killed direct from their home cages 2 h after conditioning. For the retrieval condition, animals were re-exposed to the conditioned context 48 h after conditioning for 2 min only, a manipulation we have shown produces a fear response but is not sufficient to engage extinction.^30^ For the extinction condition, animals were re-exposed to the conditioned context 48 h later for 10 min, a manipulation that is sufficient to develop robust behavioural extinction of the original context-fear association.^30^ To generate a control group for the conditioning procedure, balanced for overall stimulus exposure, we utilised dorsal hippocampal brain-derived neurotrophic factor (BDNF) antisense infusions to block contextual fear conditioning.^31^ Control rats (*n*=8) received bilateral microinfusions of 2 nmol μl^−1^ BDNF antisense oligonucleotides (5′-TCTTCCCCTTTTAATGGT-3′) into the dorsal hippocampus, sufficient to block fear conditioning, whereas rats in the consolidation group (*n*=8) received BDNF missense oligonucleotides (5′ -ATACTTTCTGTTCTTGCC-3′) at 1 μl per hemisphere into the dorsal hippocampus (AP −3.50, relative to bregma). Infusions were given 90 min before placement into the conditioning context. To generate a control condition for the retrieval group balanced for stimulus exposure, we similarly used antisense infusions previously shown to block processes that support the maintenance of the contextual fear memory, but not conditioning.^31^ Specifically, control rats (*n*=8) received dorsal hippocampal microinfusions of 2 nmol μl^−1^ Zif268 antisense oligonucleotides (5′- GGTAGTTGTCCATGGTGG-3′), whereas the retrieval group (*n*=8) received infusions of Zif268 missense oligonucleotides (5′-GTGTTCGGTAGGGTGTCA-3′). All infusions were 90 min before re-exposure to the context. In the extinction paradigm, to generate a control group matched for exposure, rats were initially trained to distinguish two contexts before contextual fear conditioning in one of the two contexts. After 48 h of conditioning, rats in the extinction group (*n*=6) were re-exposed for 10 min to the conditioned context whereas control animals (*n*=6) were re-exposed to the non-conditioned context. To assess contextual fear in the rats, freezing behaviour was quantified during all context exposures, as reported previously.^20, 21^ These behavioural data conformed to the expected patterns of conditioning and within session reductions of freezing responses to the conditioning context, indicative of extinction. Rats were killed by CO_2_ inhalation 2 h after conditioning (consolidation group) or 2 h after context exposure (retrieval and extinction groups) for tissue extraction. Experiments were conducted in accordance with the United Kingdom 1986 Animals (Scientific Procedures) Act (Project licenses PPL 30/2236 and PPL 30/2722).

### Microarray of hippocampal CA1 region gene expression

A full description of the microarray procedure and results can be found in the original studies^20, 21^ and are accessible through GEO Series accession number GSE66153. The dorsal CA1 region of the hippocampus was dissected and snap frozen before microarray analysis. Samples from individual rats were hybridised to separate microarrays (Affymetrix Rat Genome Array 230.2, Affymetrix, Santa Clara, CA, USA) as described previously.^20, 21^ There was no amplification of cDNA before hybridisation. Raw intensity data from each of the three Affymetrix data sets (consolidation, retrieval and extinction) were normalised using RMA and MAS5.0 procedures.^21^

### Gene ranking and gene set selection

Rat genes targeted by Affymetrix probe sets were identified from the Affymetrix Rat Genome Array 230.2 resources. Probe sets that do not target a unique gene were filtered from the data sets. Rat Entrez gene IDs were converted to human homologues using their shared HomoloGene ID (accessed through the MGI Vertebrate Homology table 3 March 2015). Genes that did not have a unique human homologue were excluded from the data sets. For each independent consolidation, retrieval and extinction microarray data set, MAS5.0 and RMA moderate *t*-test *P*-values, representing the significance of differential expression (increased or decreased), were combined using Fisher's method. In cases where several Affymetrix probe sets targeted the same gene, their combined *P*-values were further combined using Simes *P*-value correction procedure, giving one *P*-value per gene. For gene set selection, genes were ranked by this *P*-value. In primary analyses, the top 5% learning-related genes were taken as the gene set. For secondary analyses, the top 1, 2, 5, 10, 15, 20 and 25% genes from each data set were used. For permutation correction, the entire gene list was permuted 2000 times, each time taking the top 1–25% genes as randomised gene sets.

### Logistic regression analysis

Enrichment analysis was performed by comparing the number of consolidation-, retrieval- and extinction-related genes that overlap with case or control CNVs.^8^ For each gene set, case-control status was regressed against the number of overlapping genes and the following covariates: CNV size, number of genes per CNV, chip type and CNV study. All analyses were two-tailed. Primary analyses were corrected for multiple testing using Bonferroni's correction. Secondary analyses were corrected by calculating the fraction of randomly permuted gene sets of the same size that yielded a case enrichment as or more significant, to remove the effect of background enrichment (Supplementary Figure 1). The relative contribution of individual genes to a gene set's enrichment in case CNVs was determined by performing the same logistic regression analysis upon each gene independently. Since this was a secondary, exploratory comparison of individual gene enrichment, no *P*-value correction was performed.

## Results

### Schizophrenia CNV gene set enrichment analyses

**Figure 1:**
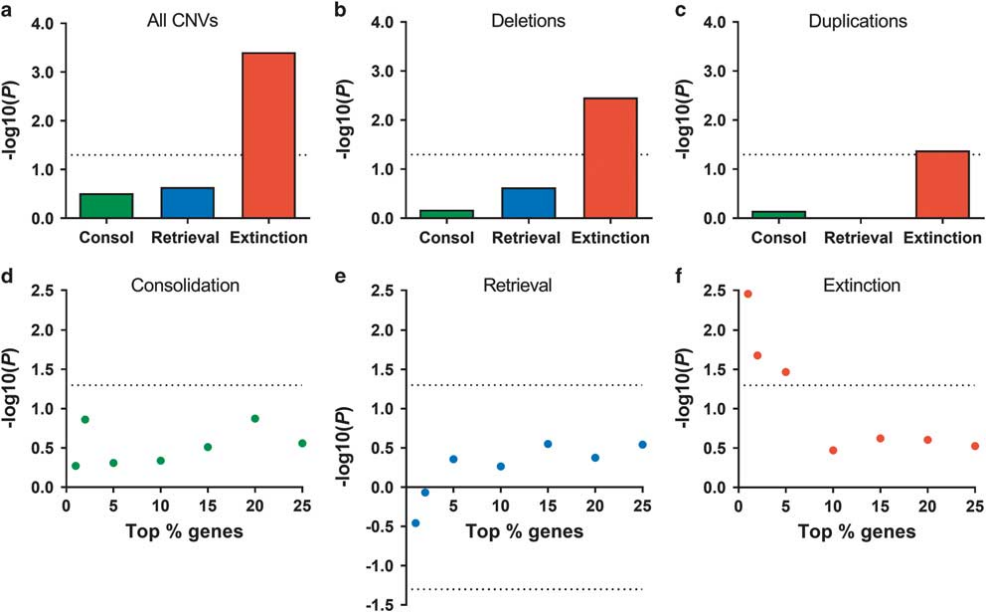
Extinction-related genes are enriched in copy number variants (CNVs) from patients with schizophrenia. (**a**) The top 5% genes differentially expressed following fear extinction (Supplementary Table 3), but not consolidation (Consol) or retrieval, are overrepresented in schizophrenia CNVs vs control CNVs (*P*=3.9 × 10^−4^). Bars represent −log_10_(*P*-value) after logistic regression enrichment analysis and Bonferroni *P*-value correction. (**b**) Deletions from schizophrenic patients alone show an overrepresentation of fear extinction-related genes (*P*=0.0034, Bonferroni corrected). (**c**) The enrichment of fear extinction-related genes in duplications from schizophrenic patients is significant (*P*=0.042, Bonferroni corrected) yet weaker than in deletions. (**d–f**) Enrichment analyses performed over the top 1, 2, 5, 10, 15, 20 and 25% genes associated with consolidation, retrieval and extinction of fear memory. Points represent −log_10_(*P*-value) × sgn(*Z*-value) where *P* is empirically determined from permutation correction (Supplementary Figure 1). For (**a–f**), dotted lines represent a *P*=0.05 threshold for statistical significance.

Consolidation, retrieval and extinction of contextual fear memory each affected the expression of a distinct group of genes in the hippocampal CA1 region, with little overlap across the learning conditions^20, 21^ (Supplementary Figure 2). We therefore investigated whether the top 5% genes differentially regulated at any one of these stages of associative learning (Supplementary Tables 1–3) are statistically overrepresented amongst genes hit by CNVs from patients with schizophrenia when compared with CNVs from controls. Using a logistic regression analysis,^8^ we found a significant enrichment of extinction-related genes in patient CNVs (extinction *P*=3.9 × 10^−4^, Bonferroni corrected, Figure 1). No such enrichment was seen for consolidation- or retrieval-related genes (consolidation *P*=0.31; retrieval *P*=0.23; Figure 1). Secondary analyses confirmed that the association of extinction-related genes with schizophrenia CNVs was robust to variation in the threshold used to define the gene sets (Figure 1). We further confirmed that the enrichment of the top 1% extinction-related genes in schizophrenia case CNVs was present independently in each of the three individual cohorts used in the analysis (ISC *P*=0.041, MGS *P*=0.029 and CLOZUK *P*=6.7 × 10^−4^), indicating that this enrichment is a general property of schizophrenia CNVs and is not driven by one population or cohort (Supplementary Table 4). The top 5% extinction-related genes were independently enriched in CNVs from the CLOZUK study (*P*=3.0 × 10^−^^4^), but not in CNVs from MGS (*P*=0.18) or ISC (*P*=0.11), which reflects the lower power of analyses in individual, smaller cohorts.

These results indicate that people with schizophrenia are enriched for CNVs affecting genes involved in extinction learning. To further investigate the origin of this effect, we examined the enrichment of learning-related genes in deletion CNVs and duplication CNVs separately. This analysis revealed that the top 5% extinction-related genes were enriched in both deletions (*P*=0.0034, Bonferroni corrected) and duplications (*P*=0.042, Bonferroni corrected; Figure 1), whereas the top 5% consolidation- and retrieval-related genes were not enriched in either CNV type.

### Control CNV enrichment analysis

To test whether the enrichment of extinction-related genes is specific to schizophrenia-associated CNVs, we conducted a control analysis using CNVs from an independent sample of 2416 patients with melanoma vs 6335 control subjects. No association was identified between consolidation-, retrieval- or extinction-related genes and melanoma case CNVs (consolidation *P*=0.59; retrieval *P*=1.0; extinction *P*=1.0; Supplementary Table 5), confirming that the enrichment of extinction-related genes in schizophrenia CNVs is a selective effect and is unlikely to reflect a general property of disease-associated CNVs.

### Genetic overlap between extinction learning and schizophrenia risk-loci

We finally sought to investigate which specific CNV-associated genes drive the enrichment for genes differentially regulated during extinction learning. The full gene set is listed in Supplementary Table 3. We note that a number of the genes driving the association with extinction learning reside within recurrent CNVs implicated in schizophrenia including *CLDN5, MFI2*, *ATP10A*, *SNRPN* and *CGNL1* (Supplementary Table 6).^4, 28^ We also identified a number of overlapping genes whose biological function remains to be well characterised, illustrating that our combined bioinformatics and functional analysis can identify the contribution to risk of genes that are not annotated sufficiently for traditional pathway analysis approaches.

## Discussion

Our results demonstrate a selective impact of genes in schizophrenia-associated CNVs on the molecular pathways regulated by extinction learning. By using gene expression data sets generated from functional experiments in animals we extend previous results from pathway analyses of schizophrenia-associated CNVs^8, 9^ and studies of individual risk genes and familial risk,^32, 33, 34^ and provide evidence that CNVs confer vulnerability to the disorder by impacting on specific cognitive processes.

Our results are consistent with previous studies suggesting that impaired extinction and inhibitory learning contribute to the development of psychotic symptoms in schizophrenia.^14, 26, 35^ Indeed, an impairment in extinction learning could explain core symptoms of the disorder, such as the characteristic persistence of delusional beliefs in schizophrenia in the face of conflicting evidence.^15^ Our findings are also consistent with previous work showing impaired extinction of fear associations in schizophrenia as well as persistent activation of the hippocampus to fearful stimuli in the disorder.^26, 36, 37^ The observation that genes in schizophrenia-associated CNVs are enriched for targets engaged during extinction learning in the CA1 region of the hippocampus also suggests a potential mechanistic explanation for the heightened activation of the CA1 region seen in both prodromal cases and individuals with established schizophrenia.^36, 38, 39^

Extinction and inhibitory learning are implicated in a number of other psychiatric and neurodevelopmental disorders. Is it notable that CNVs conferring risk to schizophrenia also confer risk for other neurodevelopmental disorders,^7, 40^ raising the possibility that the link between CNVs and associative learning is of wider relevance to those psychiatric disorders in which CNVs have a role. We also note that although we show strong evidence of an association of schizophrenia-related CNVs with extinction learning, the experimental design makes it more difficult to conclusively reject any association with consolidation and retrieval phases of associative learning.

The present results highlight the potential of using bioinformatics to integrate human genetic studies with experimental studies in model organisms in order to investigate the functional processes impacted by genetic variation. This approach overcomes some of the limitations of traditional pathway analyses, which are constrained by the prior annotation of genes and gene variants to functional pathways. The method used here, in contrast, allows all genes to be included in the analysis, regardless of their degree of prior functional annotation. The integration of human genetic data with experiments in animal models is also highly complementary to human post mortem gene expression studies, allowing additional hypothesis testing about the functional effects of risk-associated genetic variation.

Overall, these findings are consistent with the hypothesis that altered associative learning contributes to the pathogenesis of schizophrenia. Furthermore, they suggest that schizophrenia-associated CNVs particularly impact on inhibitory forms of learning such as extinction. Deficits in extinction learning may contribute to the development of both the cognitive and psychotic symptoms seen in the condition. The present results also highlight a novel approach to investigate the functional processes impacted by complex genetic risk factors in neuropsychiatric disorders by using bioinformatics approaches to integrate functional studies in animals with human genetic data.
