# Supplementary material for: Schizophrenia copy number variants and associative learning
Source: Mol Psychiatry. 2016 Dec 13;22(2):178–82. doi: 10.1038/mp.2016.227 (PMC5285462; doi:10.1038/mp.2016.227)
Supplement: Supplementary Figures, Legends and Tables 4–6 [file mp2016227x1.docx]

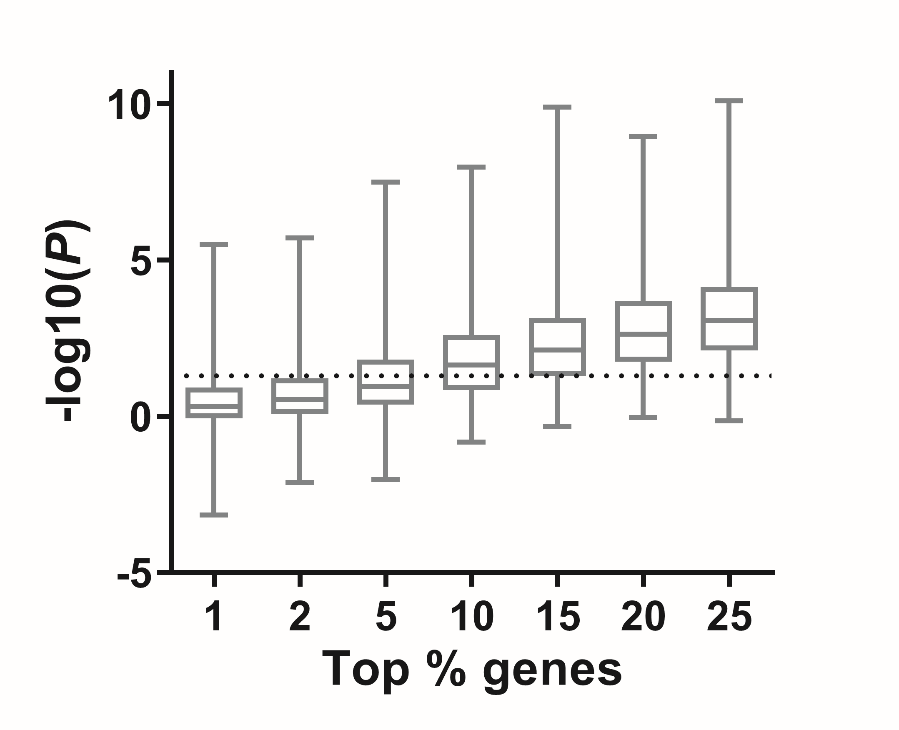


**Supplementary Figure 1** The statistical association of a gene set with case CNVs was proportional to the gene set size. All genes were permuted 2000 times, each time yielding random gene sets from the top 1, 2, 5, 10, 15, 20 and 25%. This data was used to correct *P*-values obtained from the secondary analyses of the study for background association. Boxplots display the minimum, first quartile, median, third quartile and maximum -log_10_(*P*-value) $\times$ sgn(*Z*-value) following logistic regression analyses. Dotted line represents -log_10_(*P*) where *P=*0.05.

28

34

1

35

**Supplementary Figure 2** Distinct groups of genes are associated with different components of fear memory processing. Represented here is the overlap of the top 5% genes by significance of differential expression in the CA1 region of the hippocampus two hours after each learning phase. Only one gene was common to all three gene sets (*ICOS*). The degree of overlap is no greater than chance levels (consolidation vs retrieval *P*=0.85, consolidation vs extinction *P*=0.39, retrieval vs extinction *P*=0.29 in Fisher’s exact test).

SupplementaryTable1.xlsx legend

**Supplementary Table 1** Consolidation-related genes. Listed are the top 5% (607) consolidation-related genes ranked by significance of differential expression in the rat CA1 2 hours after contextual fear conditioning^20,21^. Affymetrix IDs are from the Affymetrix Rat Genome Array version 230.2. “Expression *P*-value” relates to the significance of a gene’s differential expression following fear conditioning compared to control.

SupplementaryTable2.xlsx legend

**Supplementary Table 2** Retrieval-related genes. Listed are the top 5% (607) retrieval-related genes ranked by significance of differential expression in the rat CA1 2 hours after fear memory retrieval^20,21^. Affymetrix IDs are from the Affymetrix Rat Genome Array version 230.2. “Expression *P*-value” relates to the significance of a gene’s differential expression following retrieval compared to control.

SupplementaryTable3.xlsx legend

**Supplementary Table 3** Extinction-related genes. Listed are the top 5% (607) extinction-related genes ranked by significance of differential expression in the rat CA1 2 hours after the extinction of contextual fear memory^21^. Affymetrix IDs are from the Affymetrix Rat Genome Array version 230.2. “Expression *P*-value” relates to the significance of a gene’s differential expression following extinction learning compared to control.

| Extinction gene set | CLOZUK  *P* | MGS  *P* | ISC  *P* |
| --- | --- | --- | --- |
| Top 1% | 0.00067 | 0.029 | 0.041 |
| Top 2% | 0.0056 | 0.11 | 0.043 |
| Top 5% | 0.00030 | 0.18 | 0.11 |

**Supplementary Table 4** The enrichment of extinction-related genes in patient CNVs exists in each cohort independently. The top 1, 2 and 5% extinction-related genes were analysed for enrichment in each CNV dataset using logistic regression analysis. Sample sizes: CLOZUK 6307 cases, 10 675 controls; MGS 2215 cases, 2556 controls; ISC 3395 cases, 3185 controls.

| Gene set | All CNVs | | Deletions | | | Duplications | |
| --- | --- | --- | --- | --- | --- | --- | --- |
|  | *P* | *P*_adj_ | *P* | *P*_adj_ | *P* | | *P*_adj_ |
| Consolidation | 0.20 | 0.59 | 0.62 | 1.0 | 0.17 | | 0.50 |
| Retrieval | 0.50 | 1.0 | 0.34 | 1.0 | 0.68 | | 1.0 |
| Extinction | 0.75 | 1.0 | 0.09 | 0.28 | 0.56 | | 1.0 |

**Supplementary Table 5** There is no association of consolidation-, retrieval- or extinction-related gene sets with CNVs from patients with melanoma. The top 5% genes were analysed for enrichment in melanoma-associated CNVs vs controls using logistic regression analysis. Results are given for the combined analysis of all CNVs and for the analysis of deletions and duplications separately. *P*-values were adjusted using Bonferroni correction (*P*_adj_). Sample size: 2416 cases, 6335 controls.

| Gene symbol | Gene ID | Region | Mutation type | Reference |
| --- | --- | --- | --- | --- |
| ATP10A | 57194 | 15q11.2-13.3 | Duplication | 4 |
| CGNL1 | 84952 | 15q21.3 | Duplication | 28 |
| CLDN5 | 7122 | 22q11.2 | Deletion | 4 |
| MFI2 | 4241 | 3q29 | Deletion | 4 |
| SNRPN | 6638 | 15q11.2-13.3 | Duplication | 4 |

**Supplementary Table 6** Extinction-related genes within loci previously implicated in schizophrenia through CNV studies. Genes were taken from the top 5% extinction-related gene set. Single gene associations reported in studies indicated, using CNV datasets analysed herein. Gene symbols, gene IDs (Entrez) and regions listed pertain to the human genome. See Supplementary Table 3 for all extinction-related genes in this gene set.

**Supplementary Acknowledgements**

The authors acknowledge the contribution of data from the Database of Genotypes and Phenotypes (dbGaP): A) Genetic Epidemiology of COPD (COPDGene) Funded by the National Heart, Lung, and Blood Institute. dbGaP Study Accession: phs000179.v3.p2. The principal investigators were James D Crapo (National Jewish Health, Denver, CO, USA) and Edwin K Silverman (Brigham and Women's Hospital, Boston, MA, USA). The study was run at the National Heart, Lung and Blood Institute, Bethesda, MD, USA and funded by the National Institutes of Health, Bethesda, MD USA (U01HL089897, U01HL089856). B) A Genome-Wide Association Study of Fuchs' Endothelial Corneal Dystrophy (FECD). dbGaP Study Accession: phs000421.v1.p. The principal investigators were Natalie Afshari (Duke University, Durham, NC, USA), John Gottsch (Johns Hopkins University, Baltimore, MD, USA), Sudha K Iyengar (Case Western Reserve University, Cleveland, OH, USA), Nicholas Katsanis (Johns Hopkins University, Baltimore, MD, USA), Gordon Klintworth (Duke University, Durham, NC, USA) and Jonathan Lass (Case Western Reserve University, Cleveland, OH, USA). Co-investigators were Simon Gregory (Duke University, Durham, NC, USA) and Yi-Ju Li (Duke University, Durham, NC, USA). The study was funded by the National Eye Institute, National Institutes of Health, Bethesda, MD, USA (R01EY016482, CWRU, PI: Sudha Iyengar; R01EY016514, DUEC, PI: Gordon Klintworth; R01EY016835, JHU, PI: John Gottsch). Genotyping was carried out at the Center for Inherited Disease Research (CIDR), Johns Hopkins University, Baltimore, MD, USA and was funded by the National Institutes of Health, Bethesda, MD, USA. HHSN268200782096C, NIH contract "High throughput genotyping for studying the genetic contributions to human disease"; HHSN268201100011I, NIH contract "High throughput genotyping for studying the genetic contributions to human disease". C) California Pacific Medical Center Research Breast Health Cohort. dbGaP Study Accession: phs000395.v1.p1. The principal investigator was Elad Ziv (University of California, San Francisco, CA, USA). Co-investigators were Steven Cummings (California Pacific Medical Center Research Institute and University of California, San Francisco, CA, USA), Karla Kerlikowske (University of California, San Francisco, CA, USA) and John Shepherd (University of California, San Francisco, CA, USA). The study was run at the National Cancer Institute, National Institutes of Health, Bethesda, MD, USA and was funded by the National Institutes of Health, Bethesda, MD, USA (P01 CA107584; R01 CA120120). Genotyping was carried out at Johns Hopkins University Center for Inherited Disease Research (CIDR), Baltimore, MD, USA and was funded by the National Institutes of Health, Bethesda, MD, USA (HHSN268200782096C, "NIH contract High throughput genotyping for studying the genetic contributions to human disease"; HHSN268201100011I, "NIH contract High throughput genotyping for studying the genetic contributions to human disease"). D) Study of Melanoma Risk in Australia and the United Kingdom. dbGaP Study Accession: phs000519.v1.p1. The principal investigator was Nicholas Hayward (Queensland Institute of Medical Research, Brisbane, QLD, Australia). The study was funded by the National Cancer Institute of the National Institutes of Health, Bethesda, MD, USA (R01CA088363). Genotyping was carried out at the Center for Inherited Disease Research (CIDR), Johns Hopkins University, Baltimore, MD, USA and funded by the National Institutes of Health, Bethesda, MD, USA (HHSN268201100011I). E) Genome-Wide Association of Schizophrenia Study, (GAIN). dbGaP accession phs000021.v3.p2. Funding for this study was provided by the National Institute of Mental Health (R01 MH67257, R01 MH59588, R01 MH59571, R01 MH59565, R01 MH59587, R01 MH60870, R01 MH59566, R01 MH59586, R01 MH61675, R01 MH60879, R01 MH81800, U01 MH46276, U01 MH46289 U01 MH46318, U01 MH79469, and U01 MH79470) and the genotyping of samples was provided through the Genetic Association Information Network. The principle investigator was Pablo V. Gejman, Evanston Northwestern Healthcare (ENH) and Northwestern University, Evanston, IL, USA. F) Genome-Wide Association of Schizophrenia Study, (MGS_nonGAIN). dbGaP accession phs000167.v1.p1. Samples and associated phenotype data for the MGS_nonGAIN study were collected under the following grants: NIMH Schizophrenia Genetics Initiative U01s: MH46276 (CR Cloninger), MH46289 (C Kaufmann), and MH46318 (MT Tsuang); and MGS Part 1 (MGS1) and Part 2 (MGS2) R01s: MH67257 (NG Buccola), MH59588 (BJ Mowry), MH59571 (PV Gejman), MH59565 (Robert Freedman), MH59587 (F Amin), MH60870 (WF Byerley), MH59566 (DW Black), MH59586 (JM Silverman), MH61675 (DF Levinson), and MH60879 (CR Cloninger). G) Genetic Architecture of Smoking and Smoking Cessation dbGAP accession phs000404.v1.p1. Funding support for genotyping, which was performed at the Center for Inherited Disease Research (CIDR), was provided by 1 X01 HG005274-01. CIDR is fully funded through a federal contract from the National Institutes of Health to The Johns Hopkins University, contract number HHSN2682007 82096C. Assistance with genotype cleaning, as well as with general study coordination, was provided by the Gene Environment Association Studies (GENEVA) Coordinating Center (U01 HG004446). Funding support for collection of datasets and samples was provided by the Collaborative Genetic Study of Nicotine Dependence (COGEND; P01 CA089392) and the University of Wisconsin Transdisciplinary Tobacco Use Research Center (P50 DA019706, P50 CA084724). H) High- Density SNP Association Analysis of Melanoma: Case–Control and Outcomes Investigation. dbGaP accession phs000187.v1.p1. Research support to collect data and develop an application to support this project was provided by 3P50CA093459, 5P50CA097007, 5R01ES011740 and 5R01CA133996. I) Genetic Epidemiology of Refractive Error in the KORA Study. dbGaP accession phs000303.v1.p1. Principal investigators: Dwight Stambolian, University of Pennsylvania, Philadelphia, PA, USA; H. Erich Wichmann, Institut fu¨r Humangenetik, Helmholtz-Zentrum Mu¨nchen, Germany, National Eye Institute, National Institutes of Health, Bethesda, MD, USA. Funded by R01 EY020483, National Institutes of Health, Bethesda, MD, USA.

Samples from the WTCCC2 study were downloaded from https://www.ebi.ac.uk/ega/ and include samples from the National Blood Donors Cohort, EGAD00000000024 and samples from the 1958 British Birth Cohort, EGAD00000000022. Funding for these projects was provided by the Wellcome Trust Case Control Consortium 2 project (085475/B/08/Z and 085475/Z/ 08/Z), the Wellcome Trust (072894/Z/03/Z, 090532/Z/09/Z and 075491/Z/04/B) and NIMH grants (MH 41953 and MH08 3094). For the CLOZUK sample, we thank Novartis for their guidance and co-operation. We also thank staff at The Doctor’s Laboratory, in particular Lisa Levett and Andrew Levett, for help and advice regarding sample acquisition. We acknowledge Kiran Mantripragada, Lesley Bates, Catherine Bresner and Lucinda Hopkins for laboratory sample management. Finally, we thank the participants and clinicians who took part in the Cardiff COGS study.
